# Measuring sexual relationship power equity among young women and young men South Africa: Implications for gender-transformative programming

**DOI:** 10.1371/journal.pone.0221554

**Published:** 2019-09-25

**Authors:** Kalysha Closson, Janan J. Dietrich, Mags Beksinska, Andrew Gibbs, Stefanie Hornschuh, Tricia Smith, Jenni Smit, Glenda Gray, Thumbi Ndung’u, Mark Brockman, Angela Kaida

**Affiliations:** 1 Faculty of Health Sciences, Simon Fraser University, Burnaby, British Columbia, Canada; 2 BC Centre for Excellence in HIV/AIDS, Vancouver, British Columbia, Canada; 3 Perinatal HIV Research Unit (PHRU), Faculty of Health Sciences, University of the Witwatersrand, Johannesburg, South Africa; 4 Maternal Adolescent and Child Health (MatCH) Research Unit (MRU), Faculty of Health Sciences, University of the Witwatersrand, Durban, South Africa; 5 South African Medical Research Council, Cape Town, South Africa; 6 HIV Pathogenesis Programme and Africa Health Research Institute, University of KwaZulu-Natal, Durban, South Africa; 7 Ragon Institute of Massachusetts General Hospital, Massachusetts Institute of Technology and Harvard University, Cambridge, MA United States of America; 8 Max Planck Institute for Infection Biology, Berlin, Germany; Washington University in St. Louis, UNITED STATES

## Abstract

**Introduction:**

Measures used to assess equitable relationship dynamics, including the sexual relationship power scale (SRPS) have previously been associated with lower HIV-risk among young women, and reduced perpetration of intimate partner violence among men. However, few studies describe how the SRPS has been adapted and validated for use within global youth sexual health studies. We examined gender-specific psychometric properties, reliability, and validity of a SRPS used within a South African youth-engaged cohort study.

**Methods:**

Young men and women (16–24 years) enrolled in community-based cohorts in Durban and Soweto (2014–2016) reporting a primary partner at 6-month follow-up completed a 13-item (strongly agree/agree/disagree/strongly disagree) South African adaptation of Pulerwitz’s SRPS (range 13–52, higher scores indicating greater sexual relationship power [SRP] equity). SRPS modifications were made using gender-specific exploratory factor analyses (EFAs), removing items with factor loadings <0.3. Cronbach alphas were conducted for full and modified scales by gender. Using modified scales, unadjusted and adjusted regression models examined associations between 1. relevant socio-demographic and relationship determinants and SRP equity, and 2. SRP equity and sexual relationship related outcomes. All models adjusted for education, age, site, and current employment.

**Results:**

235 sexually-active youth (66% women, median age = 20) were included. Mean scores across all 13 scale items were 2.71 (SD 0.30) for women and 2.70 (SD 0.4) for men. Scale Cronbach’s alphas were 0.63 for women and 0.64 for men. EFAs resulted in two gender-specific single-factor SRPS. Modified SRPS Cronbach alphas increased to 0.67 for women (8-items) and 0.70 for men (9-items). After adjusting for age, site and current employment, higher education remained associated with SRP equity across genders. In adjusted models, correlates of SRP equity included primary partnerships that were age-similar (<5 years older) and <2 years in length for women and living in Soweto and younger age for men. Greater SRP equity among women was also independently associated with no recent partner violence.

**Conclusions:**

Results highlight important gender differences in SRP equity measures and associations, highlighting the critically need for future research to examine gendered constructions of SRP equity in order to accurately develop, validate and use appropriate measures within quantitative surveys.

## Introduction

A growing body of literature has demonstrated that gender inequity, including intimate partner violence (IPV) and relationships marked by sexual relationship power (SRP) inequity, is a major driver of HIV-risk for young women globally [1, 2]. This is of concern as approximately one in three women will experience some form of IPV in their lives [3]. Such global inequities have resulted in a growing need for, and increased attention on, efforts aimed at addressing gender inequity, including gender-based violence, IPV, and health outcomes that disproportionately affect women. Some of these efforts include achieving the Sustainable Development Goal (SDG) target 5.2 to eliminate all forms of violence against women by 2030 [4–6].

Other SDGs, including target 3.3 to end AIDS by 2030, need to also address gendered epidemics within settings highly impacted by HIV, such as South Africa. In South Africa young women aged 15–24 face HIV incidence rates are up to four times higher than young similarly aged men (2.54% vs. 0.55%, in 2012), accounting for approximately 2,000 new infections every week [7]. The disproportionate risk of HIV experienced by young women in such settings are multi-factorial. Biologically, young women are more susceptible to HIV acquisition due to the immaturity of the vaginal track and larger vaginal surface area [8]. These susceptibilities are heightened within socio-structural contexts that create barriers for young women to negotiate and engage in safe sexual practices [4].

Much of the existing literature examining the influence of gender inequity, limited safe sexual negotiation, and HIV susceptibility has been framed under Connell’s theory of Gender and Power [9–12]. Connell posits that due to patriarchy, a fundamental organising principle of society, men have more power than women [9, 13], which structures women’s and men’s practices and experiences. These power inequities play out within intimate relationships through men’s controlling behaviours over decisions affecting their female partners, including decisions around safe sex negotiation. SRP inequities influence women’s HIV-risk through a number of indirect and direct pathways [14]. Directly, rape has been previously described as a means in which men attempt to maintain power over women [15], and is a direct mode of HIV transmission globally [10, 16].

Indirect pathways have been found through associations between SRP inequities and increased psychological distress (e.g. depression) and substance use, which increases likelihood of engaging in behaviours (e.g. condomless sex and sex while using substances) that facilitate the passing of HIV [17–23]. SRP inequities have further been associated with experiences of IPV and reduced ability to negotiate condom use, ultimately resulting in increased risk for HIV acquisition [10, 24–28].

Sexual behaviour and relationship dynamics within South Africa are multifactorial and influenced by community, society and structural level norms and determinants, including racism, gender norms, unemployment, poverty, violence, and structural inequities [29–33]. Young women, because of the patriarchal system, may be disproportionately affected by these socio-structural inequities, including those with lower education [11]. While for young men, Connell’s [9, 13, 34] theory suggests that such structural marginalisation undermines young men’s ability to achieve respect and identity through socially approved pathways (e.g. work and providing for a family). In turn, young men may experience gender role strain [35, 36] and construct marginalized masculinities that increase HIV-risk and are predicated on control over women, perpetrating violence, having multiple concurrent partners and engaging in high levels of alcohol and substance use [37–39].

A growing number of studies have aimed to understand and act upon SRP inequities, in order to attempt to address the overlapping epidemics of gender-based violence and HIV for young women [40–44]. Intervention programs include Stepping Stones, and Stepping Stones and Creating Futures, which include a gender transformative focus that through a series of workshops attempt to address harmful gender attitudes, improve SRP equity and reduce IPV, in order to improve opportunities for young women to negotiate and successfully use condoms as a means of reducing HIV-risk [40, 45, 46].

Within quantitative research, including gender transformative programming, a majority of studies examining the determinants and outcomes related to SRP equity have used a South African modification of Pulerwitz’ 2000 SRP scale (SRPS) [11, 47]. Important details of scale reliability and validity across genders is a key knowledge gap in understanding whether or not current scales are accurately measuring the construct of SRP equity within the lives of young men and women growing up in South Africa today. Recent evidence has described the adaptation and psychometric properties of the SRPS among young Kenyan women [26], however, despite theoretical groundings acknowledging the importance of gendered power differentials, to our knowledge no data exists on how these measures have been adapted for young men in similar contexts. Moreover, no published evidence has examined the ways in which the construct of SRP, as well as scales used to measure these constructs, function differently for young South African men and women of the same age and within the same population. This includes details regarding the specific scale items used and the psychometric properties of these scales. These data gaps across studies utilizing different adaptations of the SRPS further limit cross-study comparisons, including the ability to make inferences on the effectiveness of gender transformative programming.

In order to improve SRP equity for young women and men within the context of South Africa, we need reliable and valid measures that are grounded in the lived realities of youth who are disproportionately affected by HIV. As such, there is a need to assess the validity and reliability of current metrics used to measure the construct of SRP equity among young South African women and men. In this paper we aimed to explore the gendered properties of a South African adaptation of the original SRPS [11], through three objectives including: 1) to describe gender-stratified responses and properties of the SRPS used within the youth-engaged cohort study ‘AYAZAZI’, 2) to examine differential associations between socio-demographic and relationship determinants and SRP equity and, 3) whether SRP equity is independently associated with HIV-risk, for young women and young men. Given the gendered nature of SRP equity, we expected to identify important differences in factors associated with SRP equity by gender.

## Methods

### Study setting and design

Survey data and biological samples were obtained from youth enrolled in the AYAZAZI study. AYAZAZI, meaning, “knowing themselves” in Zulu is an interdisciplinary sexual and reproductive health cohort study which assessed the multiple determinants of HIV-risk among young people living in Soweto and Durban, South Africa (aged 16–24 at baseline). AYAZAZI includes two sites; the Perinatal HIV research Unit (PHRU) located at the Chris Hani Baragwanath Hospital in Soweto and the MatCH Research Unit (MRU) based in the central business district in Durban. The two sites are particularly important in relation to the global HIV epidemic among youth, defined by the United Nations as individuals between 15 and 24 years of age [48]. Soweto, located just outside of Johannesburg in the province of Gauteng, is South Africa’s most populous black urban residential area, with 2011 Census data estimating its population to be approximately one million people living in roughly 40 separate townships [49]. While Durban, the largest city in South Africa’s KwaZulu-Natal (KZN) province, is home to approximately 20% of the country’s population at 3.4 million people [50]. In 2012, in Gauteng, HIV prevalence was 12.4%, with a prevalence 5.8% among youth aged 15–14. In the same year in KwaZulu-Natal HIV prevalence was 16.9% overall, and 12.0% among youth aged 15–24.

Participants were eligible to participate in AYAZAZI if they were 16 to 24 years of age at baseline, were HIV negative or had unknown HIV status, were not participating in another HIV prevention study, and could provide written informed consent/assent together with written parental consent (if under the age of 18) to complete the survey and undergo HIV and STI testing.

AYAZAZI was guided by a youth-engagement approach, which prioritizes the meaningful inclusion of young people at all stages of the research process [51]. From November 2014 to April 2016, trained youth research assistants recruited participants through community outreach in areas where youth frequent (e.g. clinics, shopping areas, community centres). Recruitment in Soweto was conducted within the PHRU’s HIV testing and counselling clinic, while in Durban study participants were recruited through a public clinic located near the MRU. Recruitment in Soweto occurred from November 2014 to April 2015, while Durban recruitment occurred from September 2015 to April 2016. Participants were followed-up at 3 months (Durban only), 6 months, 12 months and 18 months (Soweto only).

This study was approved by the Research Ethics Boards of Simon Fraser University (2014s0413) and the University of the Witwatersrand ([HREC]– 140707). Additional details regarding the study recruitment have been published elsewhere [52].

The questionnaire was administered by trained youth interviewers using DataFAX^TM^, and was available to participants in English, isiZulu and Sesotho. The questionnaire was developed in collaboration with South African and Canadian experts in adolescent and youth health and HIV and piloted with the study team.

### Measures

#### Outcome measure

Sexual relationship power equity was measured using a 13-item South African adolescent adaptation of Pulerwitz’ SRPS [27, 47]. The South African adaptation to the SRPS was adapted by Jewkes and colleagues in 2002 [47], and included within their 2006 evaluation of Stepping Stones, a gender transformative program aimed at reducing HIV incidence among young women [53]. Using a 4-point Likert scale, participants were asked how much they agreed or disagreed with each scale item (‘strongly agree’ to ‘strongly disagree’). Scale items were gender-specific with statements for women examining women’s perceptions of their primary partner’s controlling behaviours (e.g. “*my partner expects me to do everything for him*”), and similar to other South African studies [12, 17, 18, 54, 55], examined young men’s perceptions of their own controlling behaviour towards their primary partner (e.g. “*I expect my partner to do things for me like my ironing and cooking*”). For both young men and women, the SRPS score was summed across the 13 items with possible scores ranging from 13–52. Items denoting SRP inequities (e.g. *“My partner becomes jealous when I wear things that make me look too beautiful”*) were reversed coded so that a higher SRP score denoted greater SRP equity. Consistent with guidance from Pulerwitz et al. 2000 [11], we divided each participant’s total scale score by the number of items included in the scale, yielding a mean score for each participant. For participants with less than 2 missing responses, we imputed the median score for any missing scale responses. Note, only one missing response was noted for young women meeting the criteria to respond to the SRPS at the 6-month follow-up.

#### Covariates of SRP equity

Previous research has found associations between SRP equity and relevant social and demographic variables, as well as HIV-risk outcomes including condom use, experiences of IPV among young women, and perpetration of IPV among young men [12, 18, 26, 28, 56]. As such, we examined the content validity of modified versions of the SRPS included in the AYAZAZI study by 1) examining associations between demographic and relationship characteristics that have been previously shown to influence SRP equity, and 2) examining the relationship between SRP equity and HIV-risk outcomes.

We assessed **demographic variables,** including age (16–25 years), sexual orientation (Lesbian, gay, bisexual [LGB] vs. heterosexual), housing (formal housing vs. informal housing [reconstruction development program (RDP) housing, shack, hostel or outdoors]), personal monthly income (Less than 400ZAR, 401 to 1600ZAR, and 1601ZAR or more), and any full-time, part-time, or self-employment at time of interview. Education was assessed by asking participants if they were currently in school or not and then what was or is the highest level of education they have achieved (less than high school, completed high school only, currently in school or completed some post-secondary). Participants reported if they have ever had children and if so how many (1 or more vs. none).

**Relationship characteristics** included relationship duration with primary partner (0–11, 12–23 and ≥24 months). Primary partners were determined to be age-disparate if they were ≥5 years older than the participant [57].

#### Sexual relationship related outcomes

We assessed whether SRP equity was independently associated with two HIV-risk factors, specifically condom use at last sex with primary partner and IPV (perpetration–men; experience–women). Experiences of IPV were assessed by asking young women if they have been threatened or physically hurt by their partners in the last 6 months, and men’s reported perpetration of IPV by asking if they threatened or physically hurt any partner in the last 6 months.

### Data analysis

Of the 425 participants included in AYAZAZI, this analysis was restricted to participants who completed the 6-month follow up survey (between May 2015 and February 2016) (excluded n = 23), who reported having sex since the last visit (sexually active) (excluded n = 138), and who had a primary sexual partner in which they could reflect on while responding to items in the SRPS (excluded n = 13), resulting in a total analytical sample of 251 (59% of total cohort).

Descriptive statistics were used to summarize 6-month follow-up characteristics of the study sample overall and by gender. Differences by gender were assessed using Pearson’s Chi-square test for categorical variables and two sample Wilcoxon’s tests for continuous variables.

The mean and SD of the SRPS overall and averaged across all items were calculated among both young women and men. This average was done by summing the total scale score and dividing it by the number of items used in the scale. All analyses were stratified by the gender of respondents. Descriptive statistics were used to summarize the distribution of responses to each item in the 13-item South African youth adaptation of the SRPS.

Two exploratory factor analyses (EFAs) were performed to examine factor loadings of items in the SRPS by gender. Items with factor loadings <0.3 [26, 58] were removed and a modified scale was examined for reliability and validity. Gender-specific confirmatory factor analyses (CFAs) confirmed fit of modified models based on EFAs. Gender-specific Cronbach alphas were calculated for the total and modified versions of the SRPS included in AYAZAZI.

After checking assumptions of normality, in order to assess the content validity of the scales we used unadjusted and adjusted linear regression to examine the crude and adjusted associations between a number of a priori factors that have been previously found to be theoretically and empirically associated with SRP equity among women and men and gender specific scales, adjusting for site, education, current employment and age. If the variable of interest (e.g. education) was being tested, models adjusted for all other potential confounders (e.g. site, current employment and age). Using logistic regression, we further assessed associations between SRPS scores and condom use at last sex with primary partner, experiences of IPV (for women), and perpetration of IPV (for men), adjusting for site, current employment, education and age. Data analyses were conducted using STATA version 13 [59].

## Results

Of 251 youth included in this analysis (median age = 20, 25^th^ and 75^th^ percentile [Q1, Q3] = 16, 21), 65.3% identified as women, and 6.0% (n = 15) as LGB. The majority of participants were either currently in school or had completed some post-secondary education at time of interview (64.5%), lived in formal housing (68.9%), with a higher proportion of young men than woman (81.6% vs. 62.2%, p = 0.02). Only one-third of participants reported making more than 1601 ZAR/month (~ 125 USD), with men significantly more likely to report higher monthly income than women (41.4% vs. 27.4%, p = 0.04).

We observed important differences in 6M visit characteristics by gender (**Table 1**). Young women were more likely than young men to have children (50.6% vs. 24.4%, p<0.01), have a partner ≥5 years older than them (37.7% vs. 4.8%, p<0.001), and have been with their primary partner for ≥2 years (45.0% vs. 14.5%, p<0.01). Young men were more likely than young women to report ≥2 sexual partners since the baseline interview (50.6% vs. 10.5%, p<0.01).

**Table 1:** Comparison of socio-demographic, relationship and sexual behaviour between sexually experienced young men and women reporting a primary sexual partner (n = 251).

Variable	Total n	Overall n(%)	Young men n(%)	Young women n(%)	P-value
**Gender**	251				
Young Man		87 (34.6)			
Young Woman		164 (65.3)			
**Age** median (Q1, Q3)		20 (16–21)	19 (18–21)	20 (18.5–21)	0.04**
**Age (years)**					
17–18		70 (27.9)	29 (33.3)	41(25.0)	0.06*
19 to 21		133 (53.0)	48 (55.2)	85 (51.8)	
22 to 25		48 (19.1)	10 (11.5)	38 (23.2)	
**Site**	251				0.96
Soweto		116 (46.2)	40(46.0)	76 (46.3)	
Durban		135 (53.8)	47 (45.0)	88 (53.7)	
**Housing**	251				0.02**
Reconstruction Development Program (RDP) housing/shack/hostel or outdoors		78 (31.1)	16 (18.4)	62 (37.8)	
Formal Housing		173 (68.9)	71 (81.6)	102 (62.2)	
**Education**	251				0.263
Less than high school		27 (10.8)	12 (13.8)	15 (9.2)	
Completed high school only		62 (24.7)	17 (19.5)	45 (27.4)	
Currently in school or completed some post-secondary		162 (64.5)	58 (66.7)	104 (63.4)	
**Personal monthly Income**	251				0.04**
Less than 400 ZAR		36 (14.3)	14 (16.1)	22 (13.4)	
401–1600		134 (53.4)	37 (42.5)	97 (59.2)	
1601 or more		81 (32.3)	36 (41.4)	45 (27.4)	
**Employed at time of interview**	251				0.06*
No		180 (71.7)	56 (64.4)	124 (75.6)	
Yes		71 (28.3)	31 (35.6)	40 (24.4)	
**Sexual orientation**					0.65
Heterosexual		236 (94.0)	81 (93.1)	155 (94.5)	
Gay, Lesbian, Bisexual		15 (6.0)	6 (6.9)	9 (5.5)	
**Ever had children**	247				
No		81 (32.8)	72 (82.8)	81 (49.4)	<0.01***
Yes		166 (67.2)	21 (24.4)	83 (50.6)	
**Partner 5 years older**	246				<0.01***
Yes		65 (26.4)	4 (4.8)	61 (37.7)	
No		181(73.6)	80 (95.2)	101 (62.4)	
**Relationship length with primary partner**	**243**				<0.01***
≤11 months		81 (33.3)	53 (63.9)	28 (17.5)	
12–23 months		78 (32.1)	18 (21.7)	60 (37.5)	
≥2 years		84 (34.6)	12 (14.5)	72 (45.0)	
**Condom use at last sex with primary partner**	243				0.019**
No		93 (38.3)	23 (28.1)	70 (43.5)	
Yes		150 (61.7)	59 (72.0)	91 (56.5)	
**Experienced IPV in the last 6 months**	251				
No				151 (92.6)	
Yes				12 (7.4)	
**Perpetrated IPV in the last 6 months**	251				
No			82 (95.4)		
Yes			4 (4.7)		

*p<0.10

**p<0.05

***p<0.001

### SRP equity

Summed responses across scale items were normally distributed with mean SRP scores of 32.7 (SD 3.7) for women (**Table 2**) and 32.5 (SD 4.6) for men (**Table 3**). The average score across all items was 2.71 (SD 0.30) for women and 2.70 (SD 0.36) for men. Cronbach alphas of the 13-item SRPS was 0.63 for young women and 0.64 for young men.

**Table 2:** South African adaptation of the SRP Equity Scale item responses among sexually active young women in AYAZAZI (n = 164).

	Strongly Agree n(%)	Agree n(%)	Disagree n(%)	Strongly Disagree n(%)	Mean (SD)
1. My partner is quite comfortable when I greet men I know	2 (2.3)	42 (48.3)	36 (40.2)	8 (9.2)	2.6 (0.8)
2. My partner expects me to be at home when he comes to check me*	8 (9.1)	51 (58.0)	26 (29.6)	3 (3.4)	2.2 (0.7)
3. My partner becomes jealous when I wear things that make me look too beautiful*	9 (10.2)	36 (40.9)	34 (38.6)	9 (10.2)	2.5 (0.9)
4. My partner has more to say than I do about important decisions that affect us*	8 (9.10)	30 (34.1)	46 (52.2)	4 (4.6)	2.6 (0.8)
5. My partner never tells me who I can spend time with	18 (11.0)	56 (34.4)	81 (49.7)	8 (4.9)	2.5 (0.8)
6. I could leave our relationship any time I wanted to.	11 (12.5)	48 (54.6)	26 (29.6)	3 (3.4)	2.8 (0.8)
7. My partner does what he wants, even if I don’t want him to*	0 (0.0)	16 (18.2)	62 (70.5)	10 (11.4)	2.9 (0.6)
8. When my partner and I disagree, he gets his way most of the time*	0 (0.0)	26 (29.6)	55 (63.5)	7 (8.0)	2.8 (0.7)
9. My partner always wants to know where I am*	9 (10.2)	55(62.5)	22 (25.0)	2 (2.3)	2.2 (0.7)
10. My partner expects me to do everything for him*	0 (0.0)	13 (14.8)	67 (76.1)	8 (9.1)	3.0 (0.5)
11. Because my partner buys me things he expects me to please him*	0 (0.0)	7 (8.0)	71 (80.7)	10 (11.4)	3.1 (0.5)
12. My partner lets me know that I am not his only girlfriend*	0 (0.0)	5 (5.7)	65 (73.9)	18 (20.5)	3.3 (0.6)
13. My partner expects me to sleep over whenever he chooses*	0 (0.0)	10 (11.4)	70 (79.6)	8 (9.1)	3.1 (0.5)
Total SRPS (range 13–52, Mean [SD])					35.2 (3.8)
Average Mean (SD) across 13-items					2.7 (0.3)

*Items are reverse coded so that disagreement with the statement yielded higher equity scores

**Table 3:** South African adaptation of the SRP Equity Scale item responses among sexually active young men in AYAZAZI (n = 87).

	Strongly Agree N (%)	Agree N (%)	Disagree N (%)	Strongly Disagree N (%)	Mean (SD)
1. I am quite comfortable when my partner greets men she knows	7 (15.6)	25 (55.6)	12 (26.7)	1 (2.2)	3.0 (0.8)
2. I like my partner to be at home when I come to check her, it bothers me if she is not there*	15 (33.3)	23 (51.1)	7 (15.6)	0 (0.0)	2.0 (0.8)
3. I become jealous when my partner wears things that make her look too beautiful*	3 (6.7)	6 (13.3)	23 (51.1)	13 (28.9)	2.9 (0.9)
4. I have more to say than my partner does about important decisions that affect us*	2 (4.4)	27 (60.0)	13 (28.9)	3 (6.7)	2.5 (0.8)
5. I never tell my partner who she can see or spend time with	13 (14.9)	34 (39.1)	29 (33.3)	11 (12.6)	2.6 (0.9)
6. It might make me sad but my partner is free to leave our relationship any time she wants to	8 (17.8)	19 (42.2)	11 (24.4)	7 (15.6)	2.9 (0.9)
7. I like to do what I want, even if my partner doesn’t want me to*	6 (13.3)	19 (42.2)	12 (26.7)	8 (17.8)	2.7 (0.8)
8. When my partner and I disagree, I get my way most of the time*	2 (4.4)	25 (55.6)	15 (33.3)	3 (6.7)	2.7 (0.8)
9. I like to know where my partner is most of the time*	5 (11.1)	22 (48.9)	16 (35.6)	2 (4.4)	2.4 (0.8)
10. I expect my partner to do things for me like my ironing and cooking*	5 (11.1)	13 (28.9)	20 (44.4)	7 (15.6)	2.9 (0.9)
11. Because I buy my partner things I expect her to please me*	4 (8.9)	9 (20.0)	23 (51.1)	9 (20.0)	3.0 (0.8)
12. I let my partner know that she is not the only girlfriend I have or could have *	2 (4.4)	11 (24.4)	21 (46.7)	11 (24.4)	3.1 (0.8)
13. When I want my partner to sleep over I expect her to agree*	5 (11.1)	18 (40.0)	21 (46.7)	1 (2.2)	2.6 (0.9)
Total SRPS (range 13–52, Mean [SD])					35.1 (4.6)
Average Mean (SD) across 13-items					2.7 (0.4)

*Items are reverse coded so that disagreement with the statement yielded higher equity scores

Results from the EFAs indicated disparate factor loadings between young women and young men (see **Table 4**). Based on results from EFAs, two modified versions of the scale were created for young women and men separately. Items were removed from the 13-item scale if they had a factor loading <0.3, as recommended [11, 58] (removed non-bold items in Table 4). After examining the gender specific factor loadings only 8-items remained for young women and 9-items for young men. Of the remaining items, six were the same for young men and women. Within the gender specific modified versions of the SRPS Cronbach alphas increased to 0.67 for young women and 0.70 for young men. Responses across the modified scales remained normally distributed. Gender-specific CFAs confirmed that fit indices were significantly better using modified scales among both young men and women (data not shown).

**Table 4:** Factor loading results from Exploratory Factor Analyses (EFAs) on the full 13-item South African adaptation of the SRPS, and retained items based on EFAs by gender.

Young women (n = 164)			Young men (n = 87)		
	Factor loadings full scale	Factor loadings of retained items		Factor loadings full scale	Factor loadings of retained items
1. My partner is quite comfortable when I greet men I know	0.2444	-	**1. I am quite comfortable when my partner greets men she knows**	0.3585	0.3319
2. My partner expects me to be at home when he comes to check on me*	0.1756	-	2. I like my partner to be at home when I come to check her, it bothers me if she is not there*	0.3321	0.3430
3. My partner becomes jealous when I wear things that make me look too beautiful*	0.4700	0.3774	3. I become jealous when my partner wears things that make her look too beautiful*	0.3275	0.3382
**4. My partner has more to say than I do about important decisions that affect us**	0.3974	0.3732	4. I have more to say than my partner does about important decisions that affect us*	0.1749	-
5. My partner never tells me who I can spend time with	0.1080	-	5. I never tell my partner who she can see or spend time with	-0.0356	-
6. I could leave our relationship any time I wanted to.	0.1980	-	6. It might make me sad but my partner is free to leave our relationship any time she wants to	0.1955	-
7. My partner does what he wants, even if I don’t want him to*	0.2963	0.3211	7. I like to do what I want, even if my partner doesn’t want me to.*	0.3059	0.2970
8. When my partner and I disagree, he gets his way most of the time*	0.4576	0.4779	8. When my partner and I disagree, I get my way most of the time*	0.6062	0.5787
9. My partner always wants to know where I am*	0.1743	-	9. I like to know where my partner is most of the time*	0.5352	0.5389
10. My partner expects me to do everything for him*	0.6696	0.6592	10. I expect my partner to do things for me like my ironing and cooking*	0.5072	0.4975
11. Because my partner buys me things he expects me to please him*	0.5970	0.6260	11. Because I buy my partner things I expect her to please me*	0.4811	0.4832
12. My partner lets me know that I am not his only girlfriend*	0.5301	0.5503	12. I let my partner know that she is not the only girlfriend I have or could have *	0.1735	-
13. My partner expects me to sleep over whenever he chooses*	0.4013	0.3938	13. When I want my partner to sleep over I expect her to agree*	0.6911	0.7020

*Items were reverse coded

Items in **bold** were retained in final model (≥3 factor loading)

Of the remaining items in modified scales, 94.4% of young women and 71.1% of young men disagreed (including disagreed and strongly disagreed) to the item related to expectations of pleasing a partner if he buys her things. Just over half of young women (51.1%) agreed that her partner becomes jealous when she wears things that make her look beautiful, while only 20% of young men agreed that he became jealous when his partner wears things that make her look beautiful. Nearly all young men agreed (84.4%) that they like their partner to be at home when they check on her, and it bothers them if they are not there. Although over half of women also agreed to this statement (67.1%), this item did not load well on the scale as a whole, thus was removed from the modified scale for women.

**Table 5** shows unadjusted and adjusted associations between a number of socio-demographic, sexual behaviour, and relationship practices. In unadjusted models among women, socio-demographic factors including having a higher education, living in formal housing, having no household hunger, having no children, having a longer relationship with their primary partner, and having an age-similar primary partner were associated with greater SRP equity. After adjusting for potential confounders, only higher education, length of relationship and age difference with primary partner remained. For men, age, living in Soweto, higher education and income were associated with SRP equity in unadjusted models. In adjusted models, only younger age, site (Soweto), and higher education remained associated with SRP equity.

**Table 5:** Factors associated with higher sexual relationship power equity scores among young women (n = 164) and men (n = 87).

	Young Women		Young Men	
Variable	Unadjusted	Adjusted	Unadjusted	Adjusted
	Coefficients (β) (95%CI)	Coefficients (β) (95%CI)^	Coefficients (β) (95%CI)	Coefficients (β) (95%CI)^
**Age (per year increase)**	-0.01(-0.04 to 0.01)	0.00 (-0.02 to 0.03)	-0.05(-0.01 to -0.00)**	-0.04 (-0.09 to 0.00)*
**Site**				
Durban vs. Soweto	-0.07 (-0.17 to 0.43)	-0.01 (-0.12 to 0.10)	-0.32 (-0.50 to -0.15)***	-0.29 (-0.46 to -0.12)***
**Sexual Orientation**				
Heterosexual vs. Lesbian/Bisexual	-0.04 (-0.28 to 0.20)	-0.05 (-0.28 to 0.18)	0.05 (-0.32 to 0.43)	0.16 (-0.18 to 0.51)
**Education**				
Less than high school	Ref	Ref	Ref	
Completed high school only	0.19 (-0.01 to 0.39)*	0.19 (-0.02 to 0.39)*	0.49 (0.18 to 0.81)***	0.42 (0.12 to 0.72)***
Currently in school or completed some post-high school	0.34 (0.16 to 0.53)***	0.34 (0.15 to 0.54)***	0.45 (0.18 to 0.71)***	0.29 (0.02 to 0.72)**
**Housing**				
Formal Housing vs. RDP housing/shack/hostel or outdoors	0.14 (0.03 to 0.25)**	0.08 (-0.31 to 0.20)	-0.10 (-0.34 to 0.15)	-0.04 (-0.27 to 0.18)
**Any household hunger**				
Any vs. none	-0.13 (-0.28 to 0.02)*	-0.07 (-0.22 to 0.08)	0.07 (-0.22 to 0.35)	0.12 (-0.13 to 0.38)
**Ever had children**				
Yes vs. No	-0.14 (-0.25 to -0.03)**	-0.09 (-0.21 to 0.03)	-0.14 (-0.41 to 0.13)	-0.11 (-0.36 to 0.14)
**Employed at time of interview**				
Yes vs. No	-0.04 (-0.17 to 0.09)	0.00 (-0.13 to 0.13)	-0.10 (-0.30 to 0.09)	-0.04 (-0.23 to 0.14)
**Personal monthly Income**				
≤400ZAR	Ref	Ref	Ref	Ref
401-1600ZAR	-0.14 (-0.30 to 0.03)	-0.14 (-0.30 to 0.02)*	-1.18 (-0.45 to 0.09)	-0.03 (-0.28 to 0.23)
>1600	-0.09 (-0.27 to 0.09)	-0.09 (-0.28 to 0.10)	-0.34 (-0.61 to -0.07)**	-0.15 (-0.41 to 0.12)
**Relationship length with primary partner (n = 160)**				
≤11 months	Ref	Ref	Ref	Ref
12 to 23 months	-0.00 (-0.15 to 0.14)	0.02 (-0.13 to 0.17)	0.29 (-0.00 to 0.58)*	0.17 (-0.12 to 0.45)
≥2 years	-0.17 (-0.30 to -0.04)**	-0.14 (-0.27 to 0.00)*	0.13 (-0.14 to 0.39)	0.05 (-0.20 to 0.30)
**Age-disparate partner (≥5 years older)**	-0.11 (-0.23 to -0.00)**	-0.11 (-0.22 to -0.00)**	-0.65 (-3.49 to 2.19)	N/A

*p<0.10

**p<0.05

***p<0.001

^ Each variable adjusted for education, site, age, and employment

**Table 6** shows unadjusted and adjusted associations between SRP equity and sexual behaviour and relationship outcomes. Higher SRP equity was not associated with condom use at last sex with primary partner for either young men or young women. For young women, higher SRP equity scores was associated with reduced likelihood of experiencing IPV in the last 6 months, however SRP equity did not show an association with young men’s reported perpetration of IPV in the last 6 months.

**Table 6:** Unadjusted and adjusted association between modified SRPS and HIV-risk outcomes among young women (n = 164) and young men (n = 87).

	Condom use with primary partner at last sex		Experience of IPV in last 6 months		Perpetrated IPV in last 6 months	
	Unadjusted Coefficients(β) (95%CI)	Adjusted Coefficients(β) (95%CI)^	Unadjusted Coefficients(β) (95%CI)	Adjusted Coefficients (β) (95%CI)^	Unadjusted Coefficients(β) (95%CI)	Adjusted Coefficients (β) (95%CI)^
SRP equity young women (8-items)	0.11 (-0.10 to 0.33)	0.13 (-0.10 to 0.36)	-0.15 (-0.27 to -0.04)***	-0.14 (-0.26 to -0.01)***	-	-
SRP equity young men (9-items)	0.00 (-0.23 to 0.24)	0.06 (-0.20 to 0.31)	-	-	-0.05 (-0.16 to 0.05)	0.01 (-0.11 to 0.13)

^ All models adjusted for education, site, age, and employment

*p<0.10 **p<0.05

***p<0.001

## Discussion

Our results highlight important gender differences in the functioning of a South African SRPS used within a youth-engaged HIV prevention study. EFAs and CFAs found that items loading onto the latent construct of SRP equity were different for young men and women, resulting in two gender-specific modified scales. Using these modified scales, we further found gender-specific associations with socio-demographic, sexual behavioural and relationship characteristics. Young women and men who were currently in school or who had completed some post-secondary education had greater SRP equity compared to youth with a less than high school education. For women being in an age-disparate partnership, and being with their primary partner for two or more years (vs. less than 12 months) was associated with lower SRP equity. For young men, the only other factor associated with higher SRP equity that was not associated with SRP equity for women was living in Soweto versus Durban. SRP equity was not independently associated with condom use at last sex with primary partners for young women or young men. However, young women with lower SRP equity were more likely to have reported experiencing IPV in the last 6 months.

Of the total 13 SRPS items included in AYAZAZI, EFAs and CFAs indicated important gender differences in measuring the latent construct of SRP equity. For both young men and women many of the scale items had low factor loadings, and ‘questionable’ Cronbach alphas within the overall scales for young men and women, as well as the modified scale for women. Only the modified scale for young men resulted in an ‘acceptable’ Cronbach alpha (0.70) [60]. Low factor loadings indicate that some of the items included within the scale are potentially not accurately measuring the latent construct of SRP equity. Given that the items are being asked to a cohort of young people who are for the most part not living with their primary partners, items such as ‘I expect my partner to do things for me like ironing and cooking’, may be less relevant than for those who don’t live with their partner. It is also important to note that the wording for items across genders were at times quite different. For example, item 9 for women states “My partner always wants to know where I am” while for men states “I like to know where my partner is most of the time”. Item differences across genders, the use of absolute terms such as always and never (item 5), and cross-cultural interpretations may have resulted in participant confusion or misinterpretation. In conducting cognitive interviews on the SRPS among couples in Malawi, Conroy and colleagues found that respondents had difficulty distinguishing between real and hypothetical situations [61]. This may also be why for both men and women in our study there were low factor loadings for the statement regarding the ability to leave (young women) or for their partner to leave (young men) the relationship if they wanted.

Context specific gender norms and the terrain of gender equity in South Africa may be why we found differences in factor loadings by gender. Items surrounding jealousy (e.g. “my partner gets jealous when I wear clothes that make me look too beautiful”) had the lowest mean SRP equity scores for both men and women, indicating that jealousy may be common and an important element of SRP inequity for young people in South Africa. The two items that loaded on the scale for women but not for men tended to be related to decision-making power inequities (e.g. “My partner has more to say than I do about important decisions that affect us” and “My partner lets me know that I am not his only girlfriend”), while the three items loading for men and not women were more related to surveillance (“I like to know where my partner is most of the time”, “I like my partner to be home when I come and check on her, “it bothers me when she is not there”, and “I am quite comfortable when my partner greats men she knows”). These findings are consistent with documented gender power dynamics within South Africa that emphasise how young men who struggle to obtain socially approved ways of achieving identity, often use control in relationships with women to achieve some sense of identity [14, 62].

Differences in factor loadings, may also be related to the ways in which men and women answer questions to the items, for example, it can be noted that young women were much less likely to answer ‘strongly agree’ to any of the statements. Given these descriptive differences, the study team is currently conducting follow-up qualitative interviews with previous AYAZAZI participants to examine youth’s perspectives of scale items, and potential reasons for gender differences in factor loadings and scale reliability.

Using modified scales, we further found important gender differences in factors associated with SRP equity. For young women, many of the associations were as expected. For example, our finding that young women with a primary partner five or more years older had lower SRP equity is in line with other South African youth sexual health studies [63, 64]. Age-disparate partnerships may be more likely to be transactional in nature, where due to sexual divisions in labour and few formal employment opportunities for young women in South Africa, women seek older partners to gain material capital and social status [64]. Transactional sexual relationships may be more susceptible to reduced SRP equity, as men who are involved in transactional relationships may be more likely to exhibit controlling behaviours and perpetrate IPV [59].

As expected, we also found that women with higher SRP equity were less likely to have been threatened or physically hurt by their partner in the last 6 months. Numerous studies among young South African women have found that women with lower SRP equity were more likely to experience IPV [12, 26, 65]. However, contrary to a number of studies [28, 56, 66], we did not find an association between SRP equity and condom use with primary partners. Given that the SPRS asks specifically about respondents’ primary partners, the scale itself does not capture different forms of relationships (e.g. casual, transactional) that may have be more susceptible to SRP inequities and in turn reduced agency in condom use negotiation. In a 2016 South African study, Harrison and colleagues found that young women with higher SRP equity were less likely to use condoms. The authors hypothesize that young women with greater SRP equity may chose less risky primary sexual partners, and that within primary partnerships, the desire for intimacy and trust may influence condomless sex more than SRP inequities [67]. Future mixed-method research is needed to examine the intersections of power, intimacy, and safe sexual negotiation within a range of relationship types.

Although internal consistencies for modified scales were higher for young men than young women, few of the factors tested against the scale were found to be associated with SRP equity for young men. Results indicate that SRP equity was greater among younger aged men, those with higher education levels, and those living in Soweto compared to Durban. To our knowledge no study to date has examine socio-demographic factors associated with the SRPS among young South African men, however previous research has found associations with lower education and increased likelihood of IPV perpetration [68, 69]. Contrary to previous research among young men in sub-Saharan Africa, we did not find any independent associations between SRP equity and condom use or perpetration of IPV within primary partnerships [12, 65]. In our study, very few young men reported perpetrating IPV, as such this model had very low statistical power. As indicated by our findings, it may be the case that as men get older, they may begin to exert more controlling behaviours in their relationships. As such, future research among young South African men is needed to explore the ways in which SRP equity may function to reproduce behaviours that affect sexual relationship experiences at different ages. Moreover, programming aimed at fostering more positive gender attitudes and SRP equity for men should begin while they are young.

The 2002 South African adaptation of the SRPS was conducted among a cohort of rural youth in the province of Gauteng, and based off of a scale that was originally validated and adapted in the US in 2000 among a cohort of mainly Latina women [11]. It is likely that relationship dynamics are quite different today in a more urban setting than they were among young people in rural settings over a decade ago. The shifting ways in SRP equity plays out in young people’s relationships may be why a number of items did not load well onto the latent SRP equity variable in our study. In testing the reliability of the gender specific scales, we found higher reliability among the scale for young men. However, despite improved factor loadings of items following EFAs the Cronbach’s alphas of scales for both young men and women were moderate at best.

Despite the wide use of a South African adaptation of the SRPS [10, 18, 19, 28, 46, 55, 70–72], there is no published evidence describing the adaptation process. Within these studies there exists differences in the number of items used and no record of which items were included in the scale, making comparability across studies challenging. When using adapted versions of the SRPS as the primary exposure or outcome variable in a study we recommend that researchers include a list of the items included and detail any modifications that were made from the original scale asked within their survey. This would allow researchers to compare across studies which items may be best suited for measuring the construct of SRP equity within specific contexts. This transparency in measures should be further supplemented with additional mixed-method research that seeks to examine how SRP equity measures are perceived by young people in the current era. Creating capacity for youth insight may help to increase the internal consistency and validity of SRP equity measures within youth surveys, gender-transformative studies and programs. Furthermore, youth-engagement and insight into appropriate and relevant indicators is necessary for achieving the SDGs, including ending AIDS (SDG target 3.3) and gender-based violence (SDG target 5.2) by 2030, for young men and women within diverse contexts [73].

The findings from this exploratory study are the first to present gender-stratified descriptions of psychometric properties and validity of a SRPS for young South African women and men growing up in settings highly impacted by HIV. Although the AYAZAZI study took a youth-engaged approach, the study’s original objectives were not specifically related to SRP equity. As such, due to time and resource restraints we did not pilot and assess the SRPS with youth during the development of the survey. However, in conducting this secondary analysis on the validity of the SRPS within our cohort, we have identified a number of gaps in the current measure which we are currently exploring using qualitative follow-up interviews with AYAZAZI participants. The youth-engagement approach to the study was unique and allowed for participants and interviewers to build rapport over time, however it may also introduce some limitations. For example, reporting of sensitive questions including SRP equity, violence, and condom use may be subjective to social desirability and recall bias [74]. Given the cross-sectional nature of our study we are unable to determine a directionality of effect between SRP equity and explanatory factors. Although we adjusted our multivariate analyses for potential confounders that have been previously used to examine the validity of the SRPS within the development of the original scale [11], there may be unmeasured confounding bias affecting the results presented herein. Finally, given the use of convenience sampling within two distinct urban settings, these findings may not be generalizable to all South African youth.

This analysis is grounded in Connell’s theory of Gender and Power which acknowledges that patriarchal structures in society place many men at social and physical advantage over women [9]. As such, women face more severe consequences from experiences of IPV and consequently why we only examined the relationship between SRP equity and experiences of IPV among young women in our sample. We acknowledge that young men do face controlling behaviours, SRP inequity and IPV within their relationships, however unpacking SRP equity and experiences of IPV among young South African men is beyond the scope of this paper.

Findings from this study demonstrate the gendered nature of SRP equity and provide descriptive details into potential differences in relevant items for measuring the construct of SRP equity across genders. As such, future research needs to critically examine the gendered constructions of SRP equity, in order to accurately develop, validate and use appropriate measures within quantitative surveys. Moreover, in order to meet international targets aimed at improving the lives of young men and women globally, gendered determinants of SRP equity and pathways from SRP equity to important sexual health outcomes such as HIV need to be further explored using context specific measures informed by young people.
